# Assessing the Highest Level of Evidence from Randomized Controlled Trials in Omega-3 Research

**DOI:** 10.3390/nu15041001

**Published:** 2023-02-16

**Authors:** Sandhya Sahye-Pudaruth, David W. L. Ma

**Affiliations:** Department of Human Health and Nutritional Sciences, University of Guelph, Guelph, ON N1G 2W1, Canada

**Keywords:** cardiovascular disease (CVD), *n*-3 polyunsaturated fatty acid(*n*-3 PUFA), cancer, type 2 diabetes, acute macular degeneration (AMD), randomized controlled trials (RCTs)

## Abstract

Over the years, there has been heightened interest in the health benefits of *n*-3 polyunsaturated fatty acids (PUFA) in reducing chronic diseases such as, cardiovascular disease (CVD), cancer, type 2 diabetes, and acute macular degeneration (AMD). Due to inconsistent findings in the evidence, a review to critically examine the plethora of evidence from randomized controlled trials (RCTs) in *n*-3 PUFA research was undertaken. The aim of this review is to study the highest level of evidence and to identify gaps in *n*-3 PUFA research. RCTs were originally designed for pharmaceutical research and later adopted for nutrition and food-related research. RCTs with active diseases assume that *n*-3 PUFA will have “drug” like effects, and this high expectation may have led to the inconsistent evidence in the literature. The inconsistency in the literature may be related to varying doses of *n*-3 PUFA, sources of *n*-3 PUFA (food vs. supplement; plant vs. marine), type of *n*-3 PUFA (mixture vs. purified), trial duration, population characteristics, sample size, and genetic variation. For future research, there is a need to distinguish between primary and secondary prevention, and to focus RCTs on primary prevention of chronic diseases by *n*-3 PUFA which is lacking in the literature.

## 1. Introduction

*N*-3 long chain polyunsaturated fatty acids (*n*-3 PUFA) have an important role in human health and reduction in chronic diseases [1]. *N*-3 PUFA are presumed to have anti-inflammatory and anti-thrombotic properties, lower plasma triglycerides and low-density lipoprotein (LDL) cholesterol, improve vasomotor and endothelial functions, and inhibit cell growth factors [2,3]. There are three main types of *n*-3 PUFA, and they are alpha-linolenic acid (ALA), eicosapentaenoic acid (EPA), and docosahexaenoic acid (DHA). EPA and DHA most specifically are vital for improved cardiovascular functions, neurodevelopment, and in improving metabolic and immune processes [1]. However, it is challenging to obtain enough EPA and DHA solely from diet as they cannot be synthesized by the body and must therefore be supplemented through fish and fish oil supplements [4]. Since EPA and DHA are important for human health, numerous health organizations have recommended 250–500 mg of EPA and DHA per day [1]. While the importance of EPA and DHA for human health is recognized, there are still no established dietary reference intakes (DRIs) for EPA and DHA, though some researchers have been calling for it as this may help “inform nutrition policy decisions and reduce consumer uncertainty” [5].

Over the years, due to industrialization, there has been a reduction in the intake of *n*-3 PUFA, and increased consumption of highly processed foods rich in saturated fats and *n*-6 PUFA [6]. Consequently, a diet low in *n*-3 PUFA and high in saturated fats and *n*-6 PUFA is associated with poorer metabolic and cardiovascular health in both men and women [6]. Because of heightened interest in the health benefits of *n*-3 PUFA, there has been an increased intake of fish oil supplements among the general public, and a plethora of research on the potential effects of *n*-3 PUFA in reducing incidence of chronic and age-related diseases such as cardiovascular disease (CVD), cancer, type 2 diabetes, and acute macular degeneration (AMD) [7]. However, findings from randomized controlled trials (RCTs) studying the effects of *n*-3 PUFA supplementation on chronic disease prevention have been inconclusive, generally showing a beneficial or neutral, but not negative outcome. Additionally, the conflicting emerging evidence from newly published systematic reviews and meta-analyses (SRMA) of RCTs on the effects of *n*-3 PUFA on total CVD and its related outcomes, cancer incidence and cancer mortality has raised concerns about the role of *n*-3 PUFA in reducing and preventing chronic diseases due to challenges inherent to human studies in nutrition [8,9,10,11,12]. Since the incidence of chronic diseases is on the rise and more people are taking *n*-3 PUFA as supplements, there is a need to review the highest level of evidence from RCTs and identify study gaps in order to advance the field of *n*-3 PUFA research.

## 2. Effects of *n*-3 PUFA on Major Chronic Diseases

We initially reviewed the major findings of *n*-3 PUFA RCTs across a range of chronic diseases including CVD, cancer, diabetes, and AMD. These aforementioned diseases were the main focus of this review due to a lack of high quality RCTs in other diseases. We then examined in totality, across all these studies potential factors contributing to apparent differences in outcomes.

### 2.1. Cardiovascular Disease

Previous observational studies reported that consumption of fish once or twice per week was associated with lower risks of fatal coronary heart disease (CHD), but cohort studies are subject to confounding factors and biases and are unable to establish causality [13,14,15]. Hence, there has been an increasing interest among the scientific community to assess whether *n*-3 PUFA has a protective effect on CVD risk and its related outcomes such as myocardial infarction (MI), stroke, CHD and CVD mortality. Several trials studying the cause-and-effect relationship between *n*-3 PUFA intake and its effects on CVD and its outcomes have subsequently reported “discordant trial results” [8]. Recent evidence from SRMAs demonstrated a significant reduction in incidence of MI, CHD, total CVD, and CVD mortality after supplementation with *n*-3 PUFA [9,11,16,17]. Additionally, a dose–response meta-analysis showed that a dose of 10 g/day × years increments of *n*-3 PUFA for a longer duration was associated with a 13% reduction in major cardiovascular events [9].

Large-scale trials, with doses of *n*-3 PUFA (EPA, DHA, or EPA + DHA) ranging from 0.85 to 4 g daily and with a follow-up ranging from 3.5 to 4.9 years reported significant reductions in CVD, CHD, MI, stroke, and their related fatalities [18,19,20,21] (Table 1). The Gruppo Italiano perlo Studio della Sopravvivenza nell’Infarto Miocardico (GISSI) Prevenzione trial, an open-label RCT involving 11,324 participants with recent MI reported a 20% reduction in the primary composite of death, non-fatal MI and non-fatal stroke in the group receiving 1 g of EPA + DHA daily for 3.5 years [21]. Another open-label RCT, the GISSI-Heart Failure (HF) trial with 7046 patients with evidence of heart failure significantly reduced CVD mortality by 8% after a supplementation of 0.85–0.88 g of EPA + DHA every day for almost 4 years [19]. The Japan EPA Lipid Intervention Study (JELIS), an open-label trial conducted among 18,645 hypercholesterolemic patients further reduced major coronary events by 19% after a supplementation of 1.8 g of EPA and 5–10 mg of statin daily for 4.6 years [18]. A recent randomized, double-blind, placebo-controlled trial, the Reduction of Cardiovascular Events with Icosapent Ethyl-Intervention Trial (REDUCE-IT), reported a 25% reduction in the primary composite endpoint of cardiovascular death, nonfatal myocardial infarction (MI), nonfatal stroke, coronary revascularization, or unstable angina after a daily intake of 4 g of EPA daily for a median follow-up of 4.9 years [20].

In contrast, eleven large-scale trials with doses of *n*-3 PUFA ranging from 0.84 to 4 g daily and with a follow-up of 1 to 7.4 years reported no significant association with CVD and its sub types [22,23,24,25,26,27,28,29,30,31,32] (Table 1). Two of these large-scale trials, The Outcome Reduction with an Initial Glargine Intervention (ORIGIN) [25] and a Study of Cardiovascular Events in Diabetes (ASCEND) [29] studied the effects of *n*-3 PUFA on cardiovascular outcomes in patients with impaired fasting glucose or with diabetes, but without evidence of atherosclerotic CVD. Both had more than 12,000 participants enrolled, taking 0.84 g of DHA + EPA for more than 6 years, and still failed to show any significant reduction in CVD and its related outcomes [25,29]. Moreover, the Vitamin D and Omega-3 Trial (VITAL), a study of 25,871 subjects with no previous CVD risk factors taking 0.84 g EPA + DHA with 2000 IU of vitamin D daily for more than 5 years also reported no significant reduction in major cardiovascular events or mortality [30]. Likewise, two other large-scale trials, namely the Long-Term Outcomes Study to Assess Statin Residual Risk with Epanova in High Cardiovascular Risk Patients with Hypertriglyceridemia (STRENGTH) and the Omega-3 fatty acids in Elderly with Myocardial Infarction (OMEMI) demonstrated null findings after combined EPA + DHA therapy [31,32]. The STRENGTH trial was even stopped early because of a low possibility of demonstrating any clinical benefits [31].

**Table 1:** Characteristics of RCTs examining the effects of *n*-3 PUFA on CVD and CVD-related outcomes.

Author, Publication	Country	Follow-Up	Sample Size; (Test/Control), Description	Number of Women, %	Age (Range, Mean)	Doses of *n*-3PUFA vs. Placebo	Outcomes (Test/Control)	Results
Marchioli 2002—GISSI-P [21]	Italy	3.5 years	11,324; (5666/5658), pts with recent MI	1665 (14.7%)	59.3 years	1 g EPA + DHA + 300 mg Vit E/d or placebo	Total CVD 556/621; MI 223/233; CVD mortality 310/370; CHD mortality 209/258; Stroke 92/77	Significant reduction in Total CVD, RR 0.80 (95% CI 0.68–0.94), *p* < 0.01; CVD mortality, RR 0.70 (95% CI 0.56–0.86), *p* < 0.001; CHD mortality, RR 0.68 (95% CI 0.53–0.88), *p* < 0.01
Yokoyama 2007—JELIS [18]	Japan	4.6 years	18,645; (9326/9319) hypercholesterolemic patients on statin	12,786 (68.6%)	61 years	1.8 g EPA + 5–10 mg statin/d or placebo (5–10 mg statin)	Total CHD (Major Coronary events) 262/324; MI 73/97; Stroke 166/162; CHD mortality 29/31; MI mortality 11/14	Significant reduction in Total CHD, HR 0.81 (95% CI 0.69–0.95), *p* = 0.011
Tavazzi 2008—GISSI-HF [19]	Italy	3.9 years	7046; (3529/3517), pts with clinical evidence of heart failure	1516 (20.5%)	67 years	0.85–0.882 g EPA + DHA or placebo (olive oil)	CVD mortality 712/765; MI 107/129; Stroke 122/103; MI mortality 307/325; Stroke mortality 50/44	Significant reduction in CVD mortality, HR 0.92 (95% CI 0.83–1.02), *p* = 0.045
Galan 2010— SU.FOL.OM3 [22]	France	4.7 years	2501; (1253/1248), pts with a history of acute coronary or ischemic event 1 year before randomization	509 (20.4%)	60.6 years	0.9 g EPA + DHA + 560 μg Folate + 3 mg vitamin B-6 + B-12 (20 μg) or placebo	Total CVD 81/76; MI 32/28; CHD 51/53; Stroke 40/43	No effect on Total CVD, HR 1.08 (95% CI 0.79–1.47)
Rauch 2010— OMEGA [23]	Germany	1 year	3818; (1925/1893), pts with MI	977 (25.6%)	64 years	1 g EPA + DHA/d or placebo (olive oil)	Total CVD 182/149; MI mortality 28/29	No effect on Total CVD, OR 1.21 (95% CI 0.96–1.52)
Kroumhout et al., 2010—Alpha Omega Trial [24]	The Netherlands	3.3 years	4837; (2404/2433), pts with MI and receiving antihypertensive, antithrombotic, and lipid-modifying therapy	1054 (21.8%)	69.1 years	2 g ALA + 0.4 g EPA + DHA/d or placebo	CVD 170/185; CVD mortality 80/82; CHD mortality 67/71	No effect on Total CVD, HR 0.92 (95% CI 0.75–1.13) and other CVD outcomes
Bosch 2012—ORIGIN Trial [25]	573 centers in 40 countries globally	6.2 years	12,537; (6281/6255) pts with impaired fasting glucose, impaired glucose tolerance or diabetes	4386 (35%)	63.5 years	0.84 g EPA + DHA or placebo (olive oil).	Total CVD 1034/1017; CVD mortality 574/581; MI 344/316; Stroke 314/336	No effect on Total CVD, HR 1.01 (95% CI 0.93–1.10), and other CVD outcomes
Roncaglioni 2013—Risk & Prevention Study [26]	Italy	5 years	12,513; (6244/6269), pts with multiple CVD risk factors	4818 (38.5%)	64 years	1 g EPA + DHA/d or placebo (olive oil)	Total CVD 733/745; CVD mortality 142/137; MI 310/324; MI mortality; 82/76	No effect on Total CVD, HR 0.98 (95% CI 0.88–1.08) and other CVD outcomes
Bonds 2014— AREDS2 [27]	USA	4.8 years	4203; (2056/2147), pts with retinal findings consistent with advanced age-related macular degeneration	2387 (56.8%)	74.3 years	1 g EPA + DHA or placebo ± 10 mg lutein + 2 mg zeaxanthin	Total CVD 183/187	No effect on Total CVD, HR 0.95 (95% CI 0.78–1.17)
Andrieu et al., 2017— MAPT [28]	France and Monaco	3 years	1525; (755/770), community–dwelling and non demented pts over 70	978 (64%)	75.3 years	1.025 g EPA + DHA/d or placebo (paraffin oil)	Total CVD (cardiac and vascular disorders) 192/164; Stroke 1/2	No effect on Total CVD and stroke
Bowman 2018— ASCEND [29]	UK	7.4 years	15,480; (7740/7740), pts with diabetes but without evidence of atherosclerotic cardiovascular disease	5796 (37.4%)	63.3 years	0.84 g EPA + DHA or placebo (olive oil)	CVD 689/712; MI 186/200; Stroke 217/214; CVD mortality 196/240; CHD mortality 100/127; Stroke mortality 35/37	No effect on Total CVD, RR 0.97 (95% 0.87–1.08) and other CVD outcomes
Bhatt 2019—REDUCE IT Trial [20]	Australia, Canada, New Zealand, South Africa, the Netherlands, and USA	4.9 years	8179; (4089/4090), pts with CVD or with diabetes and other risk factors, receiving statin therapy	2357 (28.8%)	64 years	4 g EPA/d or placebo (mineral oil)	Total CVD 705/901; MI 355/250; CVD mortality 174/213; Stroke 98/134	Significant reduction in Total CVD, HR 0.75 (0.95% CI 0.68–0.83), *p* < 0.001; MI, HR 0.69 (95% CI 0.58–0.81), *p* < 0.001; CVD mortality, HR 0.80 (95% CI 0.66–0.98), *p* = 0.03; Stroke, HR 0.72 (95% CI 0.55–0.93), *p* = 0.01
Manson 2019— VITAL [30]	USA	5.3 years	25,871; (12,933/12,938), healthy men and women (no previous history of CVD, MI, stroke)	13,085 (50.6%)	67.1 years	0.84 g EPA + DHA+ 2000 IU Vit Ds/d or placebo (corn oil)	Total CVD 386/419; Total CHD 308/370; MI 145/200; Stroke 148/142; CVD mortality 142/148; CHD mortality 37/49; MI mortality 13/26; Stroke mortality 22/30	No effect on Total CVD, HR 0.92 (95% CI 0.80–1.06) and other CVD outcomes
Nicholls 2020— STRENGTH [31]	Asia, Australia, Europe, New Zealand, North America, South Africa, South America	5 years	13,078; (6539/6539), statin-treated participants with high CVD risk, hypertriglyceridemia, and low levels of HDL-C.	4568 (34.9%)	62.5 years	4 g EPA + DHA/d or placebo (corn oil)	Total CVD 785/795; MI 218/226; Stroke 142/125; CVD mortality 228/211	No effect on Total CVD, HR 0.99 (95% CI 0.90–1.09) and other CVD outcomes
Kalstad 2020— OMEMI [32]	Norway	2 years	1027; (513/154), pts with recent acute myocardial infarction	294 (28.6%)	75 years	1.6 g EPA + DHA or placebo (corn oil)	Total CVD 108/102; MI 39/35; Stroke 17/12	No effect on Total CVD, HR 1.07 (95% CI 0.82–1.40) and other CVD outcomes

AREDS 2, Age-Related Eye Disease Study 2; ASCEND, A Study of Cardiovascular Events in Diabetes; CI, confidence interval; CVD, cardiovascular disease; CHD, coronary heart disease; d, day; DHA, docosahexaenoic acid; EPA, eicosapentaenoic acid; GISSI-P, Gruppo Italiano per lo Studio della Sopravvivenza nell’Infarto Prevenzione; GISSI-HF, Gruppo Italiano per lo Studio della Sopravvivenza nell’Infarto Heart Failure; HR, hazard ratio; HDL-C, high density lipoprotein cholesterol; IU, International Unit; JELIS, Japan EPA Lipid Intervention Study; MAPT, Multidomain, Alzheimer Preventive Trial; MI, myocardial infarction; pts, patients; PUFA, polyunsaturated fatty acids; OR, odds ratio; OMEMI, Omega-3 fatty acids in Elderly with Myocardial Infarction; ORIGIN, Outcome Reduction With Initial Glargine Intervention; RR, relative risk; REDUCE-IT, Reduction of Cardiovascular Events with Icosapent Ethyl-Intervention Trial; STRENGTH, Long-Term Outcomes Study to Assess Statin Residual Risk with Epanova in High Cardiovascular Risk Patients with Hypertriglyceridemia; SU.FOL.OM3, Supplémentation en Folates et Omega-3; VITAL, VITamin D and OmegA-3 TriaL.

### 2.2. Cancer

In 2020 alone, cancer was the leading cause of morbidity and death globally with approximately 19.3 million new cancer cases and 10 million cancer-related deaths [33]. Since cancer incidence and mortality are on the rise, it has been suggested that the intake of *n*-3 PUFA may reduce cancer risk by regulating metabolic pathways and inflammatory responses, oxidative stress, and changes in the composition of membrane affecting cell signaling pathways [34,35]. It was demonstrated in vitro and in animal studies that *n*-3 PUFA may inhibit breast cancer growth [36]. Asian countries with the highest intake of *n*-3 PUFA had a significantly lower rate of breast cancer compared to Europe and United States [37]. A greater incidence of breast cancer was further observed among Asian–American women migrating to the West due to a shift from their traditional fish-based diet to a high-fat Western diet [38]. Earlier evidence focusing on Asian populations showed a protective benefit of *n*-3 PUFA, with a 20% and 26% reduction in breast cancer risk in both Japanese and Singaporean Chinese populations, respectively [39,40]. A more recent dose–response meta-analysis demonstrated a 6% decrease in breast cancer risk for every 1% increase in circulating *n*-3 PUFA [41].

However, the evidence of *n*-3 PUFA on cancer development and cancer mortality risk remains contradictory when considering all cancer types. Epidemiological studies demonstrating significant reductions in cancer risk may have overestimated the effects of *n*-3 PUFA supplementation, leading us to believe that estimates of cancer risk need to be assessed by cancer type and it is not possible to generalize across all cancers. An earlier SRMA of observational studies showed significant associations between *n*-3 PUFA and cancer incidence, especially colorectal, lung, prostate, and breast cancer [42]. More than three decades ago, the Lyon Diet Heart Study, a trial consisting of 605 MI survivors, reported that supplementing the Mediterranean diet with a margarine rich in *n*-3 PUFA reduced cancer risk by 61% after a 4 year follow up [43]. In contrast, a current SRMA of RCTs extensively examining the high-quality evidence of the effects of *n*-3 PUFA on cancer demonstrated little or no effect on cancer diagnosis and cancer mortality [12]. Consequently, the same study reported a slight increase in prostate cancer risk after an intake of ALA, although the certainty of evidence was of low-quality [12]. Most recently, large-scale RCTs such as GISSI-P, JELIS, ORIGIN, ASCEND, and VITAL showed null effects on cancer incidence and cancer mortality after more than 3.5 years of follow-up and with doses of EPA + DHA combined ranging from 0.84–1.8 g [18,29,30,44,45,46] (Table 2). Some small scale RCTs also examining the effects of *n*-3 PUFA on cancer incidence and mortality for a mean follow-up of 1.5 years found no association with cancer outcomes [47,48,49] (Table 2).

### 2.3. Type 2 Diabetes

With more than 700 million people projected to be diagnosed with type 2 diabetes by 2045, there is a need to prevent and manage this endocrine disease because of its association with increased risks of CVD and cancer [50]. Type 2 diabetes is also associated with a variety of modifiable risk factors that can be prevented by following a healthy diet and lifestyle [51]. Thus, people are now turning to *n*-3 PUFA supplementation to manage the symptoms associated with type 2 diabetes such as elevated blood glucose and insulin resistance [52]. Over the years, animal and cell culture studies have shown that *n*-3 PUFA may prevent type 2 diabetes through anti-inflammatory properties, insulin signaling, changing cell membrane function and controlling expression of glucose metabolism genes [53]. For the past decade, there has been an influx of studies investigating the effects of *n*-3 PUFA on the prevention and management of type 2 diabetes [52]. But as observed in the case of CVD and cancer prevention and treatment, the evidence for a protective effect of *n*-3 PUFA on type 2 diabetes and its related parameters also remains inconsistent [51,52].

A SRMA of 12 RCTs studying the effects of *n*-3 PUFA on glucose control reported no reduction in fasting insulin (FINS), glycosylated hemoglobin (HbA1c), and HOMA of insulin resistance (HOMA-IR) levels [51]. Another SRMA of 25 RCTs investigating the effects of *n*-3 PUFA supplementation on type 2 diabetes treatment or prevention showed a significant reduction in fasting blood glucose and insulin resistance, but no significant effect on HbA1c was observed [52]. Trials examining the effects of *n*-3 PUFA on type 2 diabetes among those with impaired glucose tolerance or with metabolic syndrome also found no significant changes in HbA1c, fasting glucose, and insulin sensitivity with the doses of *n*-3 PUFA ranging from 0.6 to 6 g per day for a duration ranging from 8 weeks to 1 year [54,55,56,57,58] (Table 3). Conversely, other trials reported a significant reduction in fasting blood glucose, insulin resistance, and HbA1c after supplementing the diet with dosage of *n*-3 PUFA ranging from 0.41 to 3 g/day for a follow-up of 8 weeks to 1 year as well [59,60,61,62,63] (Table 3). One particular RCT conducted among patients with vitamin D deficiency reported a significant increase in HbA1c in the *n*-3 PUFA group after 8 weeks of supplementation with 0.3 g of *n*-3 PUFA daily and 50,000 IU of vitamin D weekly [64].

### 2.4. Macular Degeneration

Age-related macular degeneration (AMD) accounts for 8.7% of blindness globally in people 60 years and older, while the number of people with AMD is expected to increase to 288 million by 2040 [71]. Consumption of *n*-3 PUFA has been postulated as a potentially effective strategy to protect against AMD [72]. Evidence from prospective cohort, cross-sectional, and case-control studies has previously shown an association with intake of fatty fish consumption or *n*-3 PUFA and reduced risk of AMD [73,74,75]. A newly published dose–response meta-analysis of 11 prospective cohorts reported that an increment of 1 g of EPA + DHA daily reduced early AMD risk by 50–60% [76]. Conversely, a review examining high quality evidence from only two RCTs demonstrated that supplementation of *n*-3 PUFA for up to 5 years did not reduce the incidence or risk of progression to advanced AMD [77].

The Age-Related Eye Disease Study 2 (AREDS2), a large-scale study of 4203 participants with a mean age of 73.1 years and at risk for progression to advanced AMD, reported that 1 g of EPA + DHA combined with lutein and zeaxanthin did not reduce the progression to advanced AMD after a median follow-up of 5 years [78] (Table 4). Another large-scale trial, the VITAL study found no overall effect of *n*-3 PUFA on AMD incidence or progression among 25,871 participants with a mean age of 67.1 years after a median follow-up of 5.3 years [79] (Table 4).

## 3. Conflicting Evidence in RCTs, “The Gold Standard”

In nutrition research, RCTs are considered the “gold standard” as they are known for their robustness, high-quality evidence, and ability to establish causality between exposure and outcomes [80]. Evidence-based nutrition guidelines, public health initiatives, and nutrition health claims are based on cumulative data obtained from systematic reviews and meta-analyses of RCTs [81]. As mentioned by Musa-Veloso et al., RCTs were originally designed to “test the efficacy of pharmaceuticals”, and since then, have been “adopted” in nutrition research [81]. But RCTs in nutrition research come with their share of challenges, which are not seen in drug-related research. Recruitment and selection of study participants, methods of randomization, habitual intake of nutrients of interest by study participants, blinding of study personnel and participants, adherence and compliance to the treatment regimen, identification of a suitable comparator, baseline nutrient and health status of study participants, and statistical analyses are among the various challenges faced when conducting nutrition related RCTs [80,81].

RCTs with active diseases assume that *n*-3 PUFA will have “drug” like effects. However, very few nutrients have “drug” like effects and only under acute deficiency conditions such as scurvy, rickets and osteoporosis. *N*-3 PUFA maintains its anti-inflammatory, anti-thrombotic and anti-tumor properties through multiple mechanisms such as immunomodulation, gene expression, and oxylipin synthesis [82,83]. Perhaps there is a need to recognize that even with disease prevention-like properties, *n*-3 PUFAs act long-term and it is challenging to observe short term health benefits, especially in the context of short term acute RCTs. Therefore, it is not surprising that the general conclusion that while RCTs rigorously measure the impact of an intervention by establishing the cause–effect relationship with less bias, the research surrounding *n*-3 PUFA, and the aforementioned chronic diseases remains conflicting [84].

In this review, the evidence from RCTs examining effects of *n*-3 PUFA on CVD, cancer, type 2 diabetes, and AMD, has been thoroughly summarized and the following observations are noted. The significance in reducing CVD incidence and risk by *n*-3 PUFA studies may depend on whether the trials are investigating primary or secondary prevention; the varying doses of *n*-3 PUFA and duration across trials; the different characteristics and health status of study populations where some studies included participants with higher cardiovascular risks while others included those with lower CVD risks; the large sample size of some RCTs; the daily intake of *n*-3 PUFA, supplemental or food form of *n*-3 PUFA, as well as the genetic variation influencing the absorption of *n*-3 PUFA [8]. Furthermore, the differences in the design of the trials may contribute to the significance of the results and two major examples are the GISSI-P and JELIS as open-label trials demonstrating significant reduction in CVD outcomes.

In GISSI-P, an open-label trial with a focus on secondary prevention, the significant reduction in total CVD, CVD mortality, and CHD mortality may be because participants who were survivors of their first MI, were also adhering to intensive CVD-lowering regimens such as aspirin, beta-blockers, angiotensin enzyme inhibitors, statins, as well as following a Mediterranean diet [44]. Since the participants were also administered 300 mg of vitamin E daily, it could have potentially prevented LDL oxidation, therefore lowering CVD events [85,86]. Moreover, the intake of cholesterol-lowering medications increased from 5% at baseline to 45.5% at 42 months, which may not make its results generalizable to other populations [44].

Consistent with secondary prevention studies, JELIS and REDUCE-IT are two other large-scale trials demonstrating significant reduction in CVD events [18,20]. The substantial reduction in non-fatal coronary events by 19% in the JELIS trial was mostly attributed to the administration of “highly purified EPA” instead of EPA + DHA or fish oils [18]. The high intake of fish among Japanese as compared to Western populations may have also contributed to the significant reduction in non-fatal coronary events, highlighting country-specific differences [18,87]. Moreover, to accurately determine the fish intake of JELIS population, plasma EPA fatty acid concentrations at baseline were measured and a value of 2.9 mol% was obtained, which was relatively high compared to a value of 0.3 mol% for the average US population [18]. Therefore, the significant results obtained from the JELIS trial cannot be generalized to other populations due to the elevated plasma EPA fatty acid levels among Japanese. Additionally, both treatment groups in JELIS were prescribed a low dose of statin medications as recommended by Japan’s Ministry of Health, Labor, and Welfare, known to lower lipid levels and major coronary events [88,89]. However, since 67% of the JELIS participants were females, a lower rate of coronary events was observed as women were 2.3 times less likely than men to have an incidence of coronary events [88]. The biological effects of EPA such as lowering thrombosis risks, inflammation and arrythmia, and reduction in triglycerides may also be postulated to reduce non-fatal coronary events among the JELIS participants [18].

Furthermore, in the REDUCE-IT trial, the numerous mechanisms of *n*-3 PUFA, as well as the daily intake of 4 g of EPA may have significantly reduced ischemic events [90]. As mentioned by Bhatt et al., the “EPA-related effects” such as “aggregate contribution” may have largely reduced the incidence of total ischemic events in the group receiving EPA alone [90]. Although the population of the trial were statin-treated and had low LDL-C, they also had elevated triglyceride levels and were at high risk for ischemic events, which potentially contributed to the significant reductions in ischemic events in the EPA group [90]. The use of mineral oil versus corn oil as a comparator in the REDUCE-IT trial may have contributed to the positive results of the trial because of its negative effects on Apo-B, LDL-cholesterol, and hs-CRP levels [20,91]. Conversely, large-scale RCTs such as ASCEND, ORIGIN, Risk and Prevention, STRENGTH, and VITAL failed to demonstrate significant reductions in major cardiovascular events after a follow-up ranging from 2–7.4 years and with a dosage range of 0.84–4 g of EPA + DHA [25,26,29,30,31]. These large-scale trials examined the benefits of EPA + DHA instead of purified EPA formulations as compared to JELIS and REDUCE-IT, indicating that combining EPA and DHA may have contributed to the lack of significance in reducing CVD outcomes. EPA and DHA have different biological effects on our cardiovascular system as DHA is known to moderately increase LDL-C compared to EPA in patients with elevated TG levels [92,93,94], thereby contributing to the inconsistent findings [8]. The increased use of medications lowering CVD risks such as statins, beta-blockers, and anti-coagulants may have also diluted the potential benefits of *n*-3 PUFA [30].

As discussed in the “Cancer section”, large RCTs showed little or no effect on cancer and cancer mortality risks. Findings from large-scale trials such as ASCEND, JELIS, ORIGIN, and VITAL corroborated the null results of a recently published SRMA of RCTs by Hanson et al., [12]. Data surrounding the effects of *n*-3 PUFA on different cancers have been inconsistent and were mostly based on experimental models and epidemiological studies [95]. In line with CVD, the varying doses of *n*-3 PUFA, poor allocation concealment, lack of blinding, shorter follow-up time to detect cancer incidence, and the medications prescribed to the study participants may have contributed to the lack of effects on cancer and its related outcomes [12,45]. Moreover, most trials with cancer data were not originally designed to look at the effects of *n*-3 PUFA on cancer risk, but CVD risk, which may have further contributed to the inconsistency of the evidence [45]. Since cancer is defined as an umbrella of more than 100 types of cancer, future research should examine the effects of *n*-3 PUFA specific to each cancer type, without generalizing across all cancers in order to observe a true degree of benefit [96]. Although, most of the participants from the large-scale trials such as JELIS, ORIGIN, ASCEND, and VITAL were overall healthy, it is important to note that those participating in trials are generally more health conscious, which may have mitigated the effects of *n*-3 PUFA on cancer and its related outcomes.

In addition to influencing CVD and cancer outcomes, *n*-3 PUFAs are also known to influence parameters of type 2 diabetes such as insulin resistance (IR), fasting blood glucose (FBG), and HbA1c. Inconsistent findings in the diabetes literature may be attributed to a lack of observed changes in inflammatory responses, shorter follow-up time, smaller sample size, inadequate dosage of *n*-3 PUFA, open-label design, higher attrition rate, combined intervention, baseline plasma glucose levels, health status of the study participants, HOMA-R values, and lack of sensitive methods such as the use of euglycemic clamp to detect any changes in insulin sensitivity [52,55,56,57,63]. Studies observing beneficial effects of *n*-3 PUFA on type 2 diabetes and its parameters also used combined interventions such as *n*-3 PUFA with plant sterol or vitamin D. Both plant sterol and vitamin D are known to prevent impaired glucose regulation (IGR) progression to type 2 diabetes and enhance the insulin-regulated glucose transporter type-4 (GLUT-4), respectively, while improving blood glucose levels and insulin resistance [61,62]. Therefore, the significant results from the combined intervention may have resulted in an overestimation of the true effects of *n*-3 PUFA due to the beneficial effects of plant sterols and vitamin D.

Conversely, null effects on FPG and HbA1c were observed after supplementing diets with 1.8 g of EPA daily for 6 months in the Japanese population. This null effect was due to the mean homeostasis model assessment ratio (HOMA-R) of 1.6, which was closer to the upper limit of normal HOMA-R values in the Japanese population, and as a result prevented any further reduction in FPG and HbA1c [57]. Sawada and colleagues also noted that those randomized in the EPA group had significantly lower plasma blood glucose levels than the placebo group, therefore adding to the lack of effect [57]. As Delpino et al. mentioned, 20% of their included studies in their SRMA were not double blind, which as a result may influence the behavior and responses of the participants to the treatment [52]. Additionally, study participants with various health conditions such as impaired fasting blood glucose, metabolic syndrome, vitamin D deficiency, polycystic ovarian syndrome, and hepatis may have led to the inconclusive findings on diabetes and its related parameters.

In line with the inconsistent findings related to *n*-3 PUFA research, the long-term consumption of *n*-3 PUFA has not shown a significant reduction on the incidence and progression of AMD. After a median follow-up of approximately 5 years, no significant reduction in AMD incidence and progression to advanced AMD were observed in the intervention group receiving *n*-3 PUFA in large-scale trials such as AREDS and VITAL [78,79]. These studies had a low attrition rate, large sample size, and a high level of adherence to the treatment regimen. The lack of benefit of *n*-3 PUFAs were attributed to the duration of the trial, the form of EPA and DHA used, as well as the EPA and DHA ratio since the ratio used was mostly designed for CVD related studies [78,79]. In the AREDS study, the complex secondary randomization design may have further made it more difficult to understand the role of EPA and DHA as they were combined with lutein and zeaxanthin, as well as the AREDS formulation, which consisted of vitamin C, vitamin E, beta carotene, zinc, and copper [78]. All AREDS participants took the usual AREDS formulation, leading us to believe that there may not have been a true placebo to evaluate the true effects of *n*-3 PUFA [79]. The results from AREDS may also not be generalizable since it was conducted among a highly educated and “well-nourished” group of individuals [78]. The VITAL trial also showed null effects on AMD incidence and progression, and it may be because of its low-risk population without any prior AMD and the “under ascertainment” of AMD, thus reducing the study power [79].

## 4. Strengths and Limitations

This review has a few strengths and limitations that should be addressed. First, this was a comprehensive review where the highest level evidence from *n*-3 PUFA RCTs across chronic diseases such as CVD, cancer, type 2 diabetes, and AMD were reviewed and critically analyzed. However, a potential limitation should also be considered. The totality of evidence was not considered since our focus was on high quality and large RCTs, especially for CVD, cancer, and AMD. Nevertheless, these RCTs provide important perspectives for future studies of any size.

## 5. Conclusions

In conclusion, the evidence from RCTs examining effects of *n*-3 PUFA on CVD, cancer, type 2 diabetes and its related parameters, and AMD was dependent on the types of trials, whether they were primary or secondary prevention, trial design, varying doses and forms of *n*-3 PUFA, duration of the trials, baseline characteristics of the study participants, ethnicity, geographical locations, health status of study population, the sample size, daily intake of *n*-3 PUFA, attrition rate, and participants’ adherence to the treatment regimen. To understand the controversies and inconsistencies surrounding *n*-3 PUFA, we must therefore temper our expectations around RCTs and recognize that in general, *n*-3 PUFA is not a pharmaceutical drug. We also must recognize that *n*-3 PUFA nutrition trials are not poorly done but have limitations and that the evidence from these RCTs may be stronger than they really are. The findings from this review of RCTs and *n*-3 PUFA studies also suggest that there is a need for future research to distinguish between primary and secondary prevention, and to focus more on primary prevention of the aforementioned chronic diseases and determine whether a significant and beneficial effect of *n*-3 PUFA may be obtained. Primary prevention studies will not be conflicted by co-morbidities, the use of other prescription drugs or other significant confounders. Although the research surrounding *n*-3 PUFA and age-related chronic diseases are mostly focused on treatments with EPA and DHA combined or EPA alone, future trials of DHA monotherapy are needed to confirm the mechanistic function of DHA since EPA and DHA differ in their biological effects. Overall, the expectations from large *n*-3 PUFA RCTs have not yielded conclusive evidence, but merely reaffirmed the complexity of human based nutrition research and the need to consider the totality of all study designs and the uniqueness of many different population groups.

## Figures and Tables

**Table 2:** Characteristics of RCTs studying the effects of *n*-3 PUFA on cancer and cancer-related outcomes.

Author, Publication	Country	Follow-Up	Sample Size; (Test/Control), Description	Number of Women, %	Age (Range, Mean)	Doses of *n*-3 PUFA vs. Placebo	Outcomes (Test/Control)	Results
De Lorgeril 1998—Lyon Diet Heart Study [43]	France	4 years	605; (302/303), survivors of a first MI	185 (36.6%)	<70 years	Mediterranean diet with margarine high in *n*-3 PUFA (16.4%) + advice from cardiologist and dietitian vs. placebo (regular diet)	Total cancer 7/17	Significant reduction in Total cancer by 61%, RR 0.39 (95% CI 0.15–1.01), *p* = 0.05
GISSI-P trial 1999 [44]	Italy	3.5 years	11,324; (5666/5658), pts with recent MI	1665, (14.7%)	59.3 years	1 g EPA + DHA + 300 mg vitamin E/d or placebo	Total cancer 142/134	No effect on Total cancer
von Shacky 1999—SCIMO Trial [47]	Germany	2 years	223; (112/111), pts with stenosis > 20% in one vessel	44 (19.7%)	58.4 years	3–6 g EPA + DHA (6 g for the 1st 3 mos) and 3 g EPA + DHA (for 21 mos) or placebo (fatty acids reflecting European diet)	Stomach cancer 1/0 Bronchial carcinoma 0/1	No effect on stomach cancer and bronchial carcinoma
Raitt 2005 [49]	USA	~2 years	200; (100/100) pts with an implanted cardioverter defibrillator after a recent ventricular fibrillation	28 (14%)	62.5 years	1.8 g EPA + DHA or placebo (olive oil)	Total cancer 3/4	No effect on Total cancer
Brouwer 2006—SOFA Trial [48]	Austria, Belgium, Czech Republic, Germany, the Netherlands, Poland, Switzerland and United Kingdom	1 year	546; (273/273), pts experiencing at least one confirmed ventricular fibrillation	85 (15.6%)	61.5 years	2 g EPA + DHA + other *n*-3 PUFA/d or placebo (high oleic acid sunflower oil)	Total cancer 4/1	No effect on Total cancer
Yokoyama 2007—JELIS [18]	Japan	5 years	18,645; (9326/9319) (hypercholesterolemic patients on statin)	12,786 (68.6%)	61 years	1.8 g EPA + 5–10 mg statin/d or placebo (5–10 mg statin)	Total cancer 242/218; Breast 16/21; Colorectal 26/29; Lung 32/37; Stomach 53/37	No effect on Total cancer and other cancer outcomes
Andreeva 2012— SU.FOL.OM3 [46]	France	4.7 years	2501; (1253/ 1248) pts with a history of acute coronary or ischemic event 1 year before randomization	514 (20.6%)	60.7 years	0.6 g EPA + DHA + 560 μg folate + 3 mg vitamin B-6 + B-12 (20 μg) or placebo	Total cancer 93/81	No effect on Total cancer, HR 1.17 (95% CI 0.87–1.58)
Bordeleau 2014—ORIGIN Trial [45]	573 centers in 40 countries globally	6.2 years	12,536; (6281/6255), pts with impaired fasting glucose, impaired glucose tolerance or diabetes	4386 (35%)	64 years	0.84 g EPA + DHA or placebo (olive oil)	Total cancer 463/489; mortality 177/213; Lung cancer 76/70; Colorectal 71/75; Breast 29/27; Prostate 87/90; Melanoma 18/14; Other cancers 224/253; Any skin cancers 107/111	No effect on Total cancer, HR 0.94 (95% CI 0.83–1.07) and other cancer outcomes
Bowman 2018—ASCEND [29]	UK	7.4 years	15,480; (7740/7740), pts with diabetes but without evidence of atherosclerotic cardiovascular disease	5796 (37.4%)	63.3 years	0.84 g EPA + DHA or placebo (olive oil)	Total cancer 894/890; mortality 305/319; GI 226/251; Respiratory 104/100; Genitourinary 323/303; Hematological 94/80; Breast 103/90; Melanoma skin 55/54	No effect on Total cancer, RR 1.00 (95% CI 0.91–1.01) and other cancer outcomes
Manson 2019—VITAL [30]	USA	5.3 years	25,871; (12,933/12,938), healthy men & women (no previous history of CVD, MI, stroke)	13,085 (50.6%)	67.1 years	0.84 g EPA + DHA + 2000 IU vitamin D/d or placebo (corn oil)	Total cancer 820/797; mortality 168/173; Breast 117/129; Prostate 219/192; Colorectal 54/44	No effect on Total cancer, HR 1.03 (95% CI 0.93–1.13), *p* = 0.56 and other cancer outcomes

ASCEND, A Study of Cardiovascular Events in Diabetes; CVD, cardiovascular disease; CI, confidence interval; d, day; DHA, docosahexaenoic acid; EPA, eicosapentaenoic acid; GISSI, Gruppo Italiano per lo Studio della Sopravvivenza nell’Infarto Prevenzione; HR, hazard ratio; IU, International Unit; JELIS, Japan EPA Lipid Intervention Study; MI, myocardial infarction; mos, months; pts, patients; PUFA, polyunsaturated fatty acids; ORIGIN, Outcome Reduction With Initial Glargine Intervention; RR, relative risk; SCIMO, Study on Prevention of Coronary Atherosclerosis by Intervention with Marine Omega-3 fatty acids; SOFA, Study on Omega-3 Fatty Acids and Ventricular Arrhythmia Trial; SU.FOL.OM3, Supplémentation en Folates et Omega-3; VITAL, VITamin D and OmegA-3 Trial.

**Table 3:** Characteristics of RCTs studying the effects of *n*-3 PUFA on the parameters of type 2 diabetes.

Author, Publication	Country	Follow-Up	Sample Size; (Test/Control), Description	Number of Women, %	Age (Range, Mean)	Doses of *n*-3 PUFA vs. Placebo	Results
Sirtori 1997 [54]	Italy	6 mos	935; (470/465), pts with impaired glucose tolerance	352 (37.6%)	58.5 years	1.02–1.530 g EPA + 0.7–1.05 g DHA/d or placebo (olive oil)	No effect on fasting glucose and HbA1c
Derosa 2011 [59]	Italy	6 mos	167; (78/79), pts with dyslipidemia	87 (52.1%)	54.5 years	3 g EPA + DHA/d or placebo (capsule containing sucrose, mannitol, and mineral salts) ± diet counseling	Significant reduction in fasting blood glucose, *p* < 0.05
Rafraf 2012 [65]	Iran	8 wks	61; (30/31), women with polycystic ovary syndrome	61 (100%)	27.5 years	1.2 g EPA + DHA or placebo (paraffin oil)	Significant reduction in glucose levels (by 11.4%, *p* < 0.001)
Manning 2013 [55]	New Zealand	1 year	151; (75/76), pts with metabolic syndrome	95 (62.9%)	55.8 years	0.6 g ALA ± 100 IU vitamin E or placebo	No effect on glucose levels or insulin sensitivity
Hutchins 2013 [66]	USA	12 wks	41; obese/overweight pts with impaired fasting glucose	14 (34.1%)	58.6 years	13 g or 26 g flaxseed/d or placebo (no flaxseed supplementation)	13 g flaxseed significantly reduced insulin sensitivity and glucose levels
Soares de Oliveira 2014 [67]	Brazil	12 wks	30; (15/15), women with metabolic syndrome	30 (100%)	40.1 years	0.41 g EPA + DHA/d or placebo	Significant reduction in blood glucose, *p* < 0.05
Qin 2015 [68]	China	12 wks	70; (36/34), pts with NAFLD	19 (27.1%)	45.2 years	1.24 g EPA + DHA/d or placebo (corn oil)	Significant decrease in fasting glucose, *p* < 0.05
Clark 2016 [56]	UK	9 mos	33; (16/17), patients with impaired fasting glucose	13 (39.4%)	60 years	6 g fish oil (EPA, DHA, DPA, ALA)/d or placebo (maize oil)	No effect on total glucose and insulin sensitivity
Derosa 2016 [60]	Italy	18 mos	281; (138/143), overweight patients with impaired fasting glucose	Not reported	53.4 years	3 g EPA + DHA/d or placebo (capsule containing sucrose, mannitol, and mineral salts)	Significant decrease in glycemia and insulin resistance
Freire 2016 [69]	Brazil	12 wks	52; (25/27), pts with hepatitis C	30 (57.7%)	≥18 years	1.8 g EPA + DHA/d or placebo (soybean oil) ± diet counseling	Significant reduction in insulin resistance, *p* = 0.015
Javidi 2016 [58]	Iran	12 wks	99; (66/33), prediabetic pts	52 (52.5%)	51.9 years	20 g or 40 g flaxseed powder/d or placebo (no flaxseed supplementation)	No significant reduction in insulin resistance and fasting serum glucose
Sawada 2016 [57]	Japan	6 mos	107; (53/54), pts with impaired fasting glucose	20 (18.7%)	68.4 years	1.8 g EPA/d or placebo	No significant changes in HbA1c and fasting plasma glucose levels
Wang 2019 [61]	China	12 wks	134; (76/58), pts with impaired fasting glucose	69 (51%)	56.8 years	1.4 g EPA + DHA ± 1.7 g plant sterols or placebo (soybean powder) ± 1.7 g plant sterols	Significant reduction in fasting plasma glucose, *p* < 0.01; insulin resistance, *p* < 0.01 and HbA1c, *p* ≤ 0.05
Abbott 2020 [70]	Australia	12 wks	68; (36/32), pts with abdominal obesity	43 (63.7%)	50.9 years	0.98 g DHA + EPA/d or placebo (corn oil)	Significant reduction in insulin resistance by −0.40 units, (95%CI: −0.78, −0.02, *p* = 0.038)
Raja-Naeeni 2020 [62]	Iran	8 wks	168; (84/84), prediabetic women with hypovitaminosis D	168 (100%)	41 years	2 g *n*-3 PUFAs/d ± 25,000 IU vitamin D/wk or placebo	Significant reduction in fasting glucose and insulin, *p* < 0.05
Barham 2021 [64]	Jordan	8 wks	146; (83/63), pts with vitamin D deficiency	88 (60.3%)	36.2 years	0.3 g *n*-3 PUFAs/d ± 50,000 IU vitamin D/wk or placebo	Significant increase in HbA1c, (5.79 ± 0.34 vs. 5.41 ± 0.33, *p* < 0.001) in *n*-3 PUFA group
Diaz-Rizzolo 2021 [63]	Spain	1 year	152; (75/77), pts with impaired fasting blood glucose	67 (44.1%)	71.2 years	200 g sardines/wk or placebo (nutritional education based on ADA)	Significant reduction in insulin resistance, *p* < 0.035

ADA, American Diabetes Association; ALA, alpha-linolenic acid; CI, confidence interval; d: day; DHA, docosahexaenoic acid; EPA, eicosapentaenoic acid; HbA1c, hemoglobin A1c; IU, International Unit; mos, months; NAFLD, non-alcoholic fatty liver disease; pts, patients; PUFA, polyunsaturated fatty acids; wk/s, weeks.

**Table 4:** Characteristics of RCTs studying the effects of *n*-3 PUFA on the incidence and progression of AMD.

Author, Publication	Country	Follow-Up	Sample Size; (Test/Control), Description	Number of Women, %	Age (Range, Mean)	Doses of *n*-3 PUFA vs. Placebo	Outcomes (Test/Control)	Results
AREDS2 2013 [78]	USA	5 years	4203; (2147/2056), pts with retinal findings consistent with advanced AMD	2388 (57%)	73.1 years	1 g EPA + DHA + (10 mg lutein + 2 mg zeaxanthin) or placebo (AREDS supplements)	979/961 (Advanced AMD events)	No reduction in risk of progression to advanced AMD
Christen 2020—VITAL [79]	USA	5.3 years	25,871; (12,933/12,938), healthy men and women (no previous history of CVD, MI, stroke)	13,085 (50.6%)	67.1 years	840 mg EPA + DHA + 2000 IU vitamin D/d or placebo (corn oil)	157/167	No overall effect on AMD incidence or progression

AMD, acute macular degeneration; AREDS2, The Age-Related Eye Disease Study 2; CVD, cardiovascular disease; DHA, docosahexaenoic acid; EPA, eicosapentaenoic acid; IU, International Unit; MI, myocardial infarction; pts, patients; PUFA, polyunsaturated fatty acids; VITAL, VITamin D and OmegA-3 TriaL.

## Data Availability

Not applicable.

## References

[1] Flock M.R., Harris W.S., Kris-Etherton P.M. (2013). Long-chain omega-3 fatty acids: Time to establish a dietary reference intake. Nutr. Rev..

[2] Mozaffarian D., Wu J.H. (2011). Omega-3 fatty acids and cardiovascular disease: Effects on risk factors, molecular pathways, and clinical events. J. Am. Coll. Cardiol..

[3] Weylandt K.H., Serini S., Chen Y.Q., Su H.M., Lim K., Cittadini A., Calviello G. (2015). Omega-3 Polyunsaturated Fatty Acids: The Way Forward in Times of Mixed Evidence. Biomed. Res. Int..

[4] Tur J.A., Bibiloni M.M., Sureda A., Pons A. (2012). Dietary sources of omega 3 fatty acids: Public health risks and benefits. Br. J. Nutr..

[5] Racey M., MacFarlane A., Carlson S.E., Stark K.D., Plourde M., Field C.J., Yates A.A., Wells G., Grantham A., Bazinet R.P. (2021). Dietary Reference Intakes based on chronic disease endpoints: Outcomes from a case study workshop for omega 3’s EPA and DHA. Appl. Physiol. Nutr. Metab..

[6] Yusuf S., Reddy S., ÔUnpuu S., Anand S. (2001). Global Burden of Cardiovascular Diseases. Circulation.

[7] Lee K.H., Seong H.J., Kim G., Jeong G.H., Kim J.Y., Park H., Jung E., Kronbichler A., Eisenhut M., Stubbs B. (2020). Consumption of Fish and ω-3 Fatty Acids and Cancer Risk: An Umbrella Review of Meta-Analyses of Observational Studies. Adv. Nutr..

[8] Khan S.U., Lone A.N., Khan M.S., Virani S.S., Blumenthal R.S., Nasir K., Miller M., Michos E.D., Ballantyne C.M., Boden W.E. (2021). Effect of omega-3 fatty acids on cardiovascular outcomes: A systematic review and meta-analysis. EClinicalMedicine.

[9] Xie L., Zhen P., Wei Q., Yu F., Song S., Tong J. (2021). Effects of omega-3 polyunsaturated fatty acids supplementation for patients with cardiovascular disease risks: A dose-response meta-analysis. Am. J. Transl. Res..

[10] Aung T., Halsey J., Kromhout D., Gerstein H.C., Marchioli R., Tavazzi L., Geleijnse J.M., Rauch B., Ness A., Galan P. (2018). Associations of Omega-3 Fatty Acid Supplement Use With Cardiovascular Disease Risks: Meta-analysis of 10 Trials Involving 77 917 Individuals. JAMA Cardiol..

[11] Hu Y., Hu F.B., Manson J.E. (2019). Marine Omega-3 Supplementation and Cardiovascular Disease: An Updated Meta-Analysis of 13 Randomized Controlled Trials Involving 127 477 Participants. J. Am. Heart Assoc..

[12] Hanson S., Thorpe G., Winstanley L., Abdelhamid A.S., Hooper L. (2020). Omega-3, omega-6 and total dietary polyunsaturated fat on cancer incidence: Systematic review and meta-analysis of randomised trials. Br. J. Cancer.

[13] Kromhout D., Bosschieter E.B., de Lezenne Coulander C. (1985). The inverse relation between fish consumption and 20-year mortality from coronary heart disease. N. Engl. J. Med..

[14] Zheng J., Huang T., Yu Y., Hu X., Yang B., Li D. (2012). Fish consumption and CHD mortality: An updated meta-analysis of seventeen cohort studies. Public Health Nutr..

[15] Rochon P.A., Gurwitz J.H., Sykora K., Mamdani M., Streiner D.L., Garfinkel S., Normand S.L., Anderson G.M. (2005). Reader’s guide to critical appraisal of cohort studies: 1. Role and design. BMJ.

[16] Shen S., Gong C., Jin K., Zhou L., Xiao Y., Ma L. (2022). Omega-3 Fatty Acid Supplementation and Coronary Heart Disease Risks: A Meta-Analysis of Randomized Controlled Clinical Trials. Front. Nutr..

[17] Bernasconi A.A., Wiest M.M., Lavie C.J., Milani R.V., Laukkanen J.A. (2021). Effect of Omega-3 Dosage on Cardiovascular Outcomes: An Updated Meta-Analysis and Meta-Regression of Interventional Trials. Mayo Clin. Proc..

[18] Yokoyama M., Origasa H., Matsuzaki M., Matsuzawa Y., Saito Y., Ishikawa Y., Oikawa S., Sasaki J., Hishida H., Itakura H. (2007). Effects of eicosapentaenoic acid on major coronary events in hypercholesterolaemic patients (JELIS): A randomised open-label, blinded endpoint analysis. Lancet.

[19] Tavazzi L., Maggioni A.P., Marchioli R., Barlera S., Franzosi M.G., Latini R., Lucci D., Nicolosi G.L., Porcu M., Tognoni G. (2008). Effect of *n*-3 polyunsaturated fatty acids in patients with chronic heart failure (the GISSI-HF trial): A randomised, double-blind, placebo-controlled trial. Lancet.

[20] Bhatt D.L., Steg P.G., Miller M., Brinton E.A., Jacobson T.A., Ketchum S.B., Doyle R.T., Juliano R.A., Jiao L., Granowitz C. (2019). Cardiovascular Risk Reduction with Icosapent Ethyl for Hypertriglyceridemia. N. Engl. J. Med..

[21] Marchioli R., Barzi F., Bomba E., Chieffo C., Di Gregorio D., Di Mascio R., Franzosi M.G., Geraci E., Levantesi G., Maggioni A.P. (2002). Early protection against sudden death by *n*-3 polyunsaturated fatty acids after myocardial infarction: Time-course analysis of the results of the Gruppo Italiano per lo Studio della Sopravvivenza nell’Infarto Miocardico (GISSI)-Prevenzione. Circulation.

[22] Galan P., Kesse-Guyot E., Czernichow S., Briancon S., Blacher J., Hercberg S. (2010). Effects of B vitamins and omega 3 fatty acids on cardiovascular diseases: A randomised placebo controlled trial. BMJ.

[23] Rauch B., Schiele R., Schneider S., Diller F., Victor N., Gohlke H., Gottwik M., Steinbeck G., Del Castillo U., Sack R. (2010). OMEGA, a randomized, placebo-controlled trial to test the effect of highly purified omega-3 fatty acids on top of modern guideline-adjusted therapy after myocardial infarction. Circulation.

[24] Kromhout D., Giltay E.J., Geleijnse J.M. (2010). n–3 Fatty Acids and Cardiovascular Events after Myocardial Infarction. N. Engl. J. Med..

[25] Investigators O.T., Bosch J., Gerstein H.C., Dagenais G.R., Diaz R., Dyal L., Jung H., Maggiono A.P., Probstfield J., Ramachandran A. (2012). *n*-3 fatty acids and cardiovascular outcomes in patients with dysglycemia. N. Engl. J. Med..

[26] Risk, Prevention Study Collaborative G., Roncaglioni M.C., Tombesi M., Avanzini F., Barlera S., Caimi V., Longoni P., Marzona I., Milani V. (2013). *n*-3 fatty acids in patients with multiple cardiovascular risk factors. N. Engl. J. Med..

[27] Bonds D.E., Harrington M., Worrall B.B., Bertoni A.G., Eaton C.B., Hsia J., Robinson J., Clemons T.E., Fine L.J., Chew E.Y. (2014). Effect of Long-Chain ω-3 Fatty Acids and Lutein + Zeaxanthin Supplements on Cardiovascular Outcomes. JAMA Intern. Med..

[28] Andrieu S., Guyonnet S., Coley N., Cantet C., Bonnefoy M., Bordes S., Bories L., Cufi M.-N., Dantoine T., Dartigues J.-F. (2017). Effect of long-term omega 3 polyunsaturated fatty acid supplementation with or without multidomain intervention on cognitive function in elderly adults with memory complaints (MAPT): A randomised, placebo-controlled trial. Lancet Neurol..

[29] Group A.S.C., Bowman L., Mafham M., Wallendszus K., Stevens W., Buck G., Barton J., Murphy K., Aung T., Haynes R. (2018). Effects of *n*-3 Fatty Acid Supplements in Diabetes Mellitus. N. Engl. J. Med..

[30] Manson J.E., Cook N.R., Lee I.M., Christen W., Bassuk S.S., Mora S., Gibson H., Albert C.M., Gordon D., Copeland T. (2019). Marine *n*-3 Fatty Acids and Prevention of Cardiovascular Disease and Cancer. N. Engl. J. Med..

[31] Nicholls S.J., Lincoff A.M., Garcia M., Bash D., Ballantyne C.M., Barter P.J., Davidson M.H., Kastelein J.J.P., Koenig W., McGuire D.K. (2020). Effect of High-Dose Omega-3 Fatty Acids vs Corn Oil on Major Adverse Cardiovascular Events in Patients at High Cardiovascular Risk: The STRENGTH Randomized Clinical Trial. JAMA.

[32] Kalstad A.A., Myhre P.L., Laake K., Tveit S.H., Schmidt E.B., Smith P., Nilsen D.W.T., Tveit A., Fagerland M.W., Solheim S. (2021). Effects of *n*-3 Fatty Acid Supplements in Elderly Patients After Myocardial Infarction. Circulation.

[33] Sung H., Ferlay J., Siegel R.L., Laversanne M., Soerjomataram I., Jemal A., Bray F. (2021). Global Cancer Statistics 2020: GLOBOCAN Estimates of Incidence and Mortality Worldwide for 36 Cancers in 185 Countries. CA A Cancer J. Clin..

[34] Diggle C.P. (2002). In vitro studies on the relationship between polyunsaturated fatty acids and cancer: Tumour or tissue specific effects?. Prog. Lipid Res..

[35] Gleissman H., Johnsen J.I., Kogner P. (2010). Omega-3 fatty acids in cancer, the protectors of good and the killers of evil?. Exp. Cell Res..

[36] Engeset D., Alsaker E., Lund E., Welch A., Khaw K.-T., Clavel-Chapelon F., Thiébaut A., Chajès V., Key T.J., Allen N.E. (2006). Fish consumption and breast cancer risk. The European Prospective Investigation into Cancer and Nutrition (EPIC). Int. J. Cancer.

[37] Nindrea R.D., Aryandono T., Lazuardi L., Dwiprahasto I. (2019). Protective Effect of Omega-3 Fatty Acids in Fish Consumption Against Breast Cancer in Asian Patients: A Meta-Analysis. Asian Pac. J. Cancer Prev..

[38] Ziegler R.G., Hoover R.N., Pike M.C., Hildesheim A., Nomura A.M., West D.W., Wu-Williams A.H., Kolonel L.N., Horn-Ross P.L., Rosenthal J.F. (1993). Migration patterns and breast cancer risk in Asian-American women. J. Natl. Cancer Inst..

[39] Terry P.D., Rohan T.E., Wolk A. (2003). Intakes of fish and marine fatty acids and the risks of cancers of the breast and prostate and of other hormone-related cancers: A review of the epidemiologic evidence. Am. J. Clin. Nutr..

[40] Gago-Dominguez M., Yuan J.M., Sun C.L., Lee H.P., Yu M.C. (2003). Opposing effects of dietary *n*-3 and n-6 fatty acids on mammary carcinogenesis: The Singapore Chinese Health Study. Br. J. Cancer.

[41] Yang B., Ren X.L., Wang Z.Y., Wang L., Zhao F., Guo X.J., Li D. (2019). Biomarker of long-chain *n*-3 fatty acid intake and breast cancer: Accumulative evidence from an updated meta-analysis of epidemiological studies. Crit. Rev. Food Sci. Nutr..

[42] Zhang Y.-F., Gao H.-F., Hou A.-J., Zhou Y.-H. (2014). Effect of omega-3 fatty acid supplementation on cancer incidence, non-vascular death, and total mortality: A meta-analysis of randomized controlled trials. BMC Public Health.

[43] de Lorgeril M., Salen P., Martin J.L., Monjaud I., Boucher P., Mamelle N. (1998). Mediterranean dietary pattern in a randomized trial: Prolonged survival and possible reduced cancer rate. Arch. Intern. Med..

[44] (1999). Dietary supplementation with *n*-3 polyunsaturated fatty acids and vitamin E after myocardial infarction: Results of the GISSI-Prevenzione trial. Lancet.

[45] Bordeleau L., Yakubovich N., Dagenais G.R., Rosenstock J., Probstfield J., Chang Yu P., Ryden L.E., Pirags V., Spinas G.A., Birkeland K.I. (2014). The Association of Basal Insulin Glargine and/or *n*-3 Fatty Acids With Incident Cancers in Patients With Dysglycemia. Diabetes Care.

[46] Andreeva V.A., Touvier M., Kesse-Guyot E., Julia C., Galan P., Hercberg S. (2012). B Vitamin and/or ω-3 Fatty Acid Supplementation and Cancer. Arch. Intern. Med..

[47] von Schacky C., Angerer P., Kothny W., Theisen K., Mudra H. (1999). The effect of dietary omega-3 fatty acids on coronary atherosclerosis. A randomized, double-blind, placebo-controlled trial. Ann. Intern. Med..

[48] Brouwer I.A., Zock P.L., Camm A.J., Bocker D., Hauer R.N., Wever E.F., Dullemeijer C., Ronden J.E., Katan M.B., Lubinski A. (2006). Effect of fish oil on ventricular tachyarrhythmia and death in patients with implantable cardioverter defibrillators: The Study on Omega-3 Fatty Acids and Ventricular Arrhythmia (SOFA) randomized trial. JAMA.

[49] Raitt M.H., Connor W.E., Morris C., Kron J., Halperin B., Chugh S.S., McClelland J., Cook J., MacMurdy K., Swenson R. (2005). Fish oil supplementation and risk of ventricular tachycardia and ventricular fibrillation in patients with implantable defibrillators: A randomized controlled trial. JAMA.

[50] Saeedi P., Petersohn I., Salpea P., Malanda B., Karuranga S., Unwin N., Colagiuri S., Guariguata L., Motala A.A., Ogurtsova K. (2019). Global and regional diabetes prevalence estimates for 2019 and projections for 2030 and 2045: Results from the International Diabetes Federation Diabetes Atlas, 9th edition. Diabetes Res. Clin. Pract..

[51] Gao C., Liu Y., Gan Y., Bao W., Peng X., Xing Q., Gao H., Lai J., Liu L., Wang Z. (2020). Effects of fish oil supplementation on glucose control and lipid levels among patients with type 2 diabetes mellitus: A Meta-analysis of randomized controlled trials. Lipids Health Dis..

[52] Delpino F.M., Figueiredo L.M., Da Silva B.G.C., Da Silva T.G., Mintem G.C., Bielemann R.M., Gigante D.P. (2021). Omega-3 supplementation and diabetes: A systematic review and meta-analysis. Crit. Rev. Food Sci. Nutr..

[53] Oh P.C., Koh K.K., Sakuma I., Lim S., Lee Y., Lee S., Lee K., Han S.H., Shin E.K. (2014). Omega-3 fatty acid therapy dose-dependently and significantly decreased triglycerides and improved flow-mediated dilation, however, did not significantly improve insulin sensitivity in patients with hypertriglyceridemia. Int. J. Cardiol..

[54] Sirtori C.R., Paoletti R., Mancini M., Crepaldi G., Manzato E., Rivellese A., Pamparana F., Stragliotto E. (1997). *N*-3 fatty acids do not lead to an increased diabetic risk in patients with hyperlipidemia and abnormal glucose tolerance. Italian Fish Oil Multicenter Study. Am. J. Clin. Nutr..

[55] Manning P.J., Sutherland W.H., Williams S.M., Walker R.J., Berry E.A., De Jong S.A., Ryalls A.R. (2013). The effect of lipoic acid and vitamin E therapies in individuals with the metabolic syndrome. Nutr. Metab. Cardiovasc. Dis..

[56] Clark L.F., Thivierge M.C., Kidd C.A., McGeoch S.C., Abraham P., Pearson D.W.M., Horgan G.W., Holtrop G., Thies F., Lobley G.E. (2016). Fish oil supplemented for 9 months does not improve glycaemic control or insulin sensitivity in subjects with impaired glucose regulation: A parallel randomised controlled trial. Br. J. Nutr..

[57] Sawada T., Tsubata H., Hashimoto N., Takabe M., Miyata T., Aoki K., Yamashita S., Oishi S., Osue T., Yokoi K. (2016). Effects of 6-month eicosapentaenoic acid treatment on postprandial hyperglycemia, hyperlipidemia, insulin secretion ability, and concomitant endothelial dysfunction among newly-diagnosed impaired glucose metabolism patients with coronary artery disease. An open label, single blinded, prospective randomized controlled trial. Cardiovasc. Diabetol..

[58] Javidi A., Mozaffari-Khosravi H., Nadjarzadeh A., Dehghani A., Eftekhari M.H. (2016). The effect of flaxseed powder on insulin resistance indices and blood pressure in prediabetic individuals: A randomized controlled clinical trial. J. Res. Med. Sci..

[59] Derosa G., Cicero A.F.G., Fogari E., D’Angelo A., Bonaventura A., Maffioli P. (2011). Effects of *n*-3 PUFA on insulin resistance after an oral fat load. Eur. J. Lipid Sci. Technol..

[60] Derosa G., Cicero A.F., D’Angelo A., Borghi C., Maffioli P. (2016). Effects of *n*-3 pufas on fasting plasma glucose and insulin resistance in patients with impaired fasting glucose or impaired glucose tolerance. Biofactors.

[61] Wang J.F., Zhang H.M., Li Y.Y., Xia S., Wei Y., Yang L., Wang D., Ye J.J., Li H.X., Yuan J. (2019). A combination of omega-3 and plant sterols regulate glucose and lipid metabolism in individuals with impaired glucose regulation: A randomized and controlled clinical trial. Lipids Health Dis..

[62] Rajabi-Naeeni M., Dolatian M., Qorbani M., Vaezi A.A. (2020). The effect of omega-3 and vitamin D co-supplementation on glycemic control and lipid profiles in reproductive-aged women with pre-diabetes and hypovitaminosis D: A randomized controlled trial. Diabetol. Metab. Syndr..

[63] Diaz-Rizzolo D.A., Serra A., Colungo C., Sala-Vila A., Siso-Almirall A., Gomis R. (2021). Type 2 diabetes preventive effects with a 12-months sardine-enriched diet in elderly population with prediabetes: An interventional, randomized and controlled trial. Clin. Nutr..

[64] Barham A., Mohammad B., Hasoun L., Awwad S., Mosleh I., Aljaberi A., Abu-Samak M. (2021). The combination of omega-3 fatty acids with high doses of vitamin D3 elevate A1c levels: A randomized Clinical Trial in people with vitamin D deficiency. Int. J. Clin. Pract..

[65] Rafraf M., Mohammadi E., Asghari-Jafarabadi M., Farzadi L. (2012). Omega-3 Fatty Acids Improve Glucose Metabolism without Effects on Obesity Values and Serum Visfatin Levels in Women with Polycystic Ovary Syndrome. J. Am. Coll. Nutr..

[66] Hutchins A.M., Brown B.D., Cunnane S.C., Domitrovich S.G., Adams E.R., Bobowiec C.E. (2013). Daily flaxseed consumption improves glycemic control in obese men and women with pre-diabetes: A randomized study. Nutr. Res..

[67] Soares de Oliveira Carvalho A.P., Kimi Uehara S., Nogueria Netto J.F., Rosa G. (2014). Hypocaloric diet associated with the consumption of jam enriched with microencapsulated fish oil decreases insulin resistance. Nutr. Hosp..

[68] Qin Y., Zhou Y., Chen S.H., Zhao X.L., Ran L., Zeng X.L., Wu Y., Chen J.L., Kang C., Shu F.R. (2015). Fish Oil Supplements Lower Serum Lipids and Glucose in Correlation with a Reduction in Plasma Fibroblast Growth Factor 21 and Prostaglandin E2 in Nonalcoholic Fatty Liver Disease Associated with Hyperlipidemia: A Randomized Clinical Trial. PLoS ONE.

[69] Freire T.O., Boulhosa R.S.S.B., Oliveira L.P.M., De Jesus R.P., Cavalcante L.N., Lemaire D.C., Toralles M.B.P., Lyra L.G.C., Lyra A.C. (2016). n-3 polyunsaturated fatty acid supplementation reduces insulin resistance in hepatitis C virus infected patients: A randomised controlled trial. J. Hum. Nutr. Diet..

[70] Abbott K.A., Burrows T.L., Acharya S., Thota R.N., Garg M.L. (2020). DHA-enriched fish oil reduces insulin resistance in overweight and obese adults. Prostaglandins Leukot Essent Fat. Acids.

[71] Wong W.L., Su X., Li X., Cheung C.M.G., Klein R., Cheng C.-Y., Wong T.Y. (2014). Global prevalence of age-related macular degeneration and disease burden projection for 2020 and 2040: A systematic review and meta-analysis. Lancet Glob. Health.

[72] Akuffo K.O., Beatty S., Stack J., Dennison J., O’Regan S., Meagher K.A., Peto T., Nolan J. (2014). Central Retinal Enrichment Supplementation Trials (CREST): Design and methodology of the CREST randomized controlled trials. Ophthalmic Epidemiol..

[73] Parekh N., Chappell R.J., Millen A.E., Albert D.M., Mares J.A. (2007). Association between vitamin D and age-related macular degeneration in the Third National Health and Nutrition Examination Survey, 1988 through 1994. Arch. Ophthalmol..

[74] Smith W., Mitchell P., Leeder S.R. (2000). Dietary fat and fish intake and age-related maculopathy. Arch. Ophthalmol..

[75] Seddon J.M., Rosner B., Sperduto R.D., Yannuzzi L., Haller J.A., Blair N.P., Willett W. (2001). Dietary fat and risk for advanced age-related macular degeneration. Arch. Ophthalmol..

[76] Zhong Y., Wang K., Jiang L., Wang J., Zhang X., Xu J., Yao K. (2021). Dietary fatty acid intake, plasma fatty acid levels, and the risk of age-related macular degeneration (AMD): A dose-response meta-analysis of prospective cohort studies. Eur. J. Nutr..

[77] Lawrenson J.G., Evans J.R. (2015). Omega 3 fatty acids for preventing or slowing the progression of age-related macular degeneration. Cochrane Database Syst. Rev..

[78] Age-Related Eye Disease Study 2 Research G. (2013). Lutein + zeaxanthin and omega-3 fatty acids for age-related macular degeneration: The Age-Related Eye Disease Study 2 (AREDS2) randomized clinical trial. JAMA.

[79] Christen W.G., Cook N.R., Manson J.E., Buring J.E., Chasman D.I., Lee I.M., Bubes V., Li C., Haubourg M., Schaumberg D.A. (2020). Effect of Vitamin D and omega-3 Fatty Acid Supplementation on Risk of Age-Related Macular Degeneration: An Ancillary Study of the VITAL Randomized Clinical Trial. JAMA Ophthalmol..

[80] Lichtenstein A.H., Petersen K., Barger K., Hansen K.E., Anderson C.A.M., Baer D.J., Lampe J.W., Rasmussen H., Matthan N.R. (2021). Perspective: Design and Conduct of Human Nutrition Randomized Controlled Trials. Adv. Nutr..

[81] Musa-Veloso K., Racey M., MacFarlane A., Bier D., Lamarche B., Trumbo P., House J. (2021). Challenges in the design, interpretation, and reporting of randomized controlled clinical studies on the health effects of whole foods. Appl. Physiol. Nutr. Metab..

[82] Musazadeh V., Dehghan P., Saleh-Ghadimi S., Abbasalizad Farhangi M. (2021). Omega 3-rich Camelina sativa oil in the context of a weight loss program improves glucose homeostasis, inflammation and oxidative stress in patients with NAFLD: A randomised placebo-controlled clinical trial. Int. J. Clin. Pract..

[83] Calder P.C. (2020). Eicosapentaenoic and docosahexaenoic acid derived specialised pro-resolving mediators: Concentrations in humans and the effects of age, sex, disease and increased omega-3 fatty acid intake. Biochimie.

[84] Hariton E., Locascio J.J. (2018). Randomised controlled trials—The gold standard for effectiveness research. BJOG Int. J. Obstet. Gynaecol..

[85] Princen H.M., van Duyvenvoorde W., Buytenhek R., van der Laarse A., van Poppel G., Gevers Leuven J.A., van Hinsbergh V.W. (1995). Supplementation with low doses of vitamin E protects LDL from lipid peroxidation in men and women. Arter. Thromb. Vasc. Biol..

[86] de Waart F.G., Moser U., Kok F.J. (1997). Vitamin E supplementation in elderly lowers the oxidation rate of linoleic acid in LDL. Atherosclerosis.

[87] Nakamura T., Azuma A., Kuribayashi T., Sugihara H., Okuda S., Nakagawa M. (2003). Serum fatty acid levels, dietary style and coronary heart disease in three neighbouring areas in Japan: The Kumihama study. Br. J. Nutr..

[88] Matsuzaki M., Kita T., Mabuchi H., Matsuzawa Y., Nakaya N., Oikawa S., Saito Y., Sasaki J., Shimamoto K., Itakura H. (2002). Large scale cohort study of the relationship between serum cholesterol concentration and coronary events with low-dose simvastatin therapy in Japanese patients with hypercholesterolemia. Circ. J..

[89] Nakamura H., Arakawa K., Itakura H., Kitabatake A., Goto Y., Toyota T., Nakaya N., Nishimoto S., Muranaka M., Yamamoto A. (2006). Primary prevention of cardiovascular disease with pravastatin in Japan (MEGA Study): A prospective randomised controlled trial. Lancet.

[90] Bhatt D.L., Steg P.G., Miller M., Brinton E.A., Jacobson T.A., Ketchum S.B., Doyle R.T., Juliano R.A., Jiao L., Granowitz C. (2019). Effects of Icosapent Ethyl on Total Ischemic Events. J. Am. Coll. Cardiol..

[91] Food And Drug Administration (2016). FDA Briefing Document: Endocrine and Metabolic Drug Advisory Committee Meeting.

[92] Ballantyne C.M., Bays H.E., Kastelein J.J., Stein E., Isaacsohn J.L., Braeckman R.A., Soni P.N. (2012). Efficacy and safety of eicosapentaenoic acid ethyl ester (AMR101) therapy in statin-treated patients with persistent high triglycerides (from the ANCHOR study). Am. J. Cardiol..

[93] Bays H.E., Braeckman R.A., Ballantyne C.M., Kastelein J.J., Otvos J.D., Stirtan W.G., Soni P.N. (2012). Icosapent ethyl, a pure EPA omega-3 fatty acid: Effects on lipoprotein particle concentration and size in patients with very high triglyceride levels (the MARINE study). J. Clin. Lipidol..

[94] Davidson M.H., Stein E.A., Bays H.E., Maki K.C., Doyle R.T., Shalwitz R.A., Ballantyne C.M., Ginsberg H.N., COMBination of prescription Omega-3 with Simvastatin (COMBOS) Investigators (2007). Efficacy and tolerability of adding prescription omega-3 fatty acids 4 g/d to simvastatin 40 mg/d in hypertriglyceridemic patients: An 8-week, randomized, double-blind, placebo-controlled study. Clin. Ther..

[95] Gerber M. (2012). Omega-3 fatty acids and cancers: A systematic update review of epidemiological studies. Br. J. Nutr..

[96] National Cancer Institute What Is Cancer?. https://www.cancer.gov/about-cancer/understanding/what-is-cancer.

